# A “Ping-Pong” left atrial thrombus mimicking left atrial myxoma: A case report

**DOI:** 10.1016/j.amsu.2022.104328

**Published:** 2022-08-06

**Authors:** Erlangga Diasmara Hargiyanto, Ivana Purnama Dewi, Budi Baktijasa Dharmadjati

**Affiliations:** a Faculty of Medicine, Airlangga University, Surabaya, Indonesia; b Department of Cardiology and Vascular Medicine, Dr. Soetomo General Hospital, Surabaya, Indonesia; c Faculty of Medicine, Duta Wacana Christian University, Yogyakarta, Indonesia

**Keywords:** Mitral stenosis, Valvular heart disease, Left atrial thrombus, Atrial fibrillation, Case report

## Abstract

**Introduction:**

Valvular heart disease is highly prevalent, especially in developing countries. Mitral Stenosis (MS) is a condition where there is narrowing of mitral heart valve. Left atrial (LA) thrombus is often seen in severe MS patients.

**Case presentation:**

A 47-year-old woman complained of palpitation and shortness of breath. The heart sounded irregularly irregular, with grade III/IV diastolic murmurs at the apex. Her electrocardiogram showed atrial fibrillation (AF) with rapid ventricular response Transthoracal echocardiography (TTE) showed severe MS, mild tricuspid regurgitation, and LA thrombus. Mitral valve replacement surgery, tricuspid valve repair, and evacuation of the LA thrombus were immediately done. We evacuated a spherical mass with a size of 4 × 3x2.2 cm, layered and easily separated. Microscopic examination showed extensive fibrin and bleeding with mononuclear inflammatory cells and macrophages, corresponding to a thrombus conclusion.

**Clinical discussion:**

Atrial thrombus is common in MS patients. The incidence will increase by about two times in patients with AF. TTE is a reliable tool in diagnosing large mobile atrial thrombus and differentiated it from other cardiac masses. However, histopathological examination is still the gold standard to distinguish between LA thrombus and myxoma. Immediate thrombus evacuation and valve replacement, if needed, will give good results and reduce systemic thromboembolism.

**Conclusion:**

LA thrombus is often seen in a patient with severe MS. Optimal preoperative preparation involves assessing preoperative risk stratification will give good results.

## Highlights

• Left atrial thrombus is often seen in a patient with severe mitral stenosis with atrial fibrillation.
• Transthoracal echocardiography is an essential and simple modality to diagnose cardiac masses such as large mobile atrial thrombus or myxoma.
• Histopathological examination is the gold standard to distinguish between atrial thrombus and myxoma.
• Several scoring systems can help to assess preoperative preparation such as EuroSCORE and STS score.

## 1 Introduction

Valvular heart disease has a relatively high prevalence, especially in developing countries. The epidemiology of valvular heart disease has changed dramatically with socio-economic development and changes in the composition of the population with older mean age [1]. The most affected valves are the mitral and aortic valves [2].

Left atrial (LA) thrombus is common in patients with severe mitral stenosis (MS). On echocardiography, often found also dilatation of the LA. The prevalence of LA thrombus is about 17% in patients with severe MS and will increase by about two times in patients with atrial fibrillation [3]. The most common thrombus location is in the left atrial appendage (LAA), but sometimes it can also be in the LA [4].

This case report demonstrates that a free-moving thrombus in the LA is often the result of severe MS, with an electrocardiogram showing atrial fibrillation. Optimal preoperative preparation before evacuation of the thrombus and valve replacement, if needed, will give good results and reduce systemic thromboembolism. This case report has been reported in line with the SCARE criteria [5].

## 2 Case presentation

A 47-year-old woman came to the cardiology outpatient clinic of tertiary hospital with complaints of palpitations and shortness of breath, especially during strenuous activities. The patient also complained of swollen both legs. There was no previous diabetes mellitus, hypertension, or coronary heart disease history. The patient just found out that she had heart disease at this time. There was no history of smoking, alcohol, recreational drug use, or allergies. Neither family has ever had a disease like this before.

Physical examination obtained blood pressure of 105/79 mmHg, pulse 130 beats per minute irregular, and breathing 18 times per minute. On chest examination, heart sounds S1 S2 irregularly irregular with a grade III/IV diastolic murmur at the apex. There are no rales or wheezing in the pulmonology examination. On examination of the extremities, there is pitting edema on both legs. Laboratory examinations within normal limits. An electrocardiogram examination showed atrial fibrillation rhythm with 110–140 beats per minute rapid ventricular response. The patient was then given intravenous rate control to achieve a moderate ventricular response. Chest X-ray showed cardiomegaly with a cardiothoracic ratio of 65%. The waist of the heart is flat; the conus pulmonalis is prominent, the right border of the right heart widens to the right, and there is a double contour that supports the image of the mitral heart configuration.

**Fig. 1:**
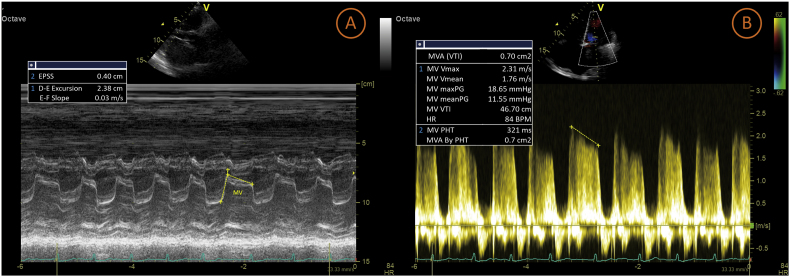
(A) The mitral valve shows slow early diastolic closure on an M-mode echocardiographic examination. The E-F slope, or mid-diastolic closure velocity, is significantly decreased or even flat, (B) Doppler Echocardiography shows severe MS.

**Fig. 2:**
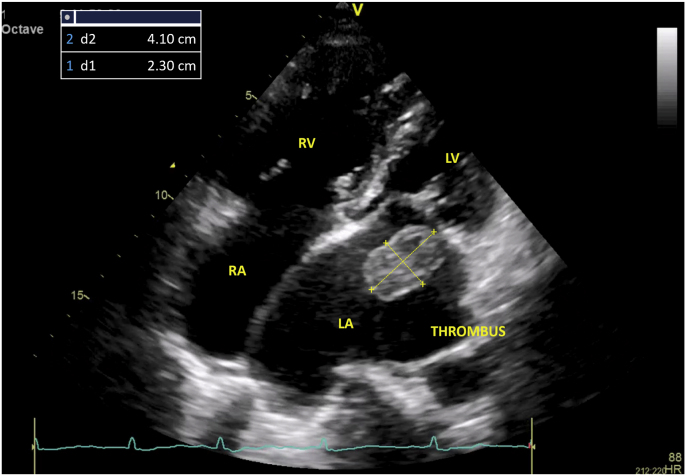
4 chamber view shows there was a ping-pong-like thrombus in LA with a size of 4.1 × 2.3 cm.

Transthoracal echocardiography (TTE) examination performed by an echocardiography expert supported the clinical diagnosis of with severe MS (MV mean PG 11.55 mmHg; MVA (VTI) 0.7 cm^2^; MV PHT 321 ms; MVA By PHT 0.7 cm^2^; MVA planimetry 0.7 cm^2^) Wilkins Score 2-2-3-3 (Fig. 1, Movie 1). There was mild mitral regurgitation (MR ERO 0.1 cm^2^; MR RV 11 ml), mild tricuspid regurgitation (TR maxPG 31.37 mmHg), and mild pulmonary regurgitation (PR Dec Slope 1.7 m/s2). Cardiac chamber dimensions showed LA dilatation (LA major 7.9cm; LA minor 6.4cm), right atrial dilatation (RA major 7.5cm; RA minor 3.5cm) with an est RAP of 15 mmHg, and right ventricular dilatation (RVDB 3.4cm) with mild pulmonary hypertension (estPASP 46.37 mmHg; PV AccT 101 ms). There was a ping-pong-like thrombus in the LA with a size of 4.1 × 2.3 cm (Fig. 2, Movie 2). LV systolic function (EF by Mod A2C 61%) and RV systolic function (TAPSE 2.1cm) were normal. Left ventricular (LV) segmental analysis showed paradoxical IVS. There is LV concentric remodeling (LVDMi 62.97 g/m2; RWT 0.468).

csSupplementary video related to this article can be found at doi:10.1016/j.amsu.2022.104328

The following is/are the supplementary data related to this article:

Movie 1 Short axis view TTE shows severe MS with impression of thrombus obstruction at MV1 Movie 1

Movie 2 4 chamber view shows there was a ping-pong-like thrombus in LA Movie 2

**Fig. 3:**
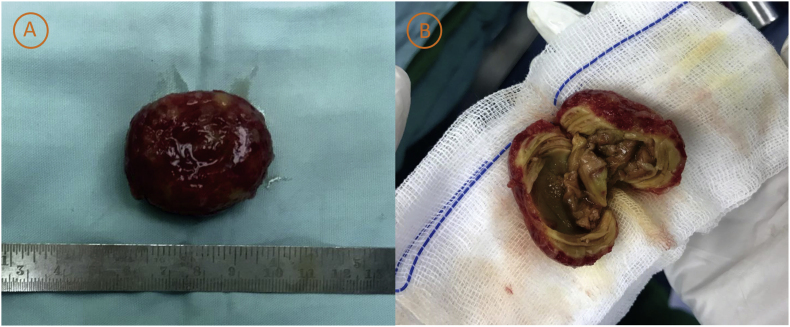
(A) Macroscopic appearance of left atrial thrombus (4 × 3 × 2.2 cm), (B) It was multi-layered with dense brownish mass in the center.

We calculate EuroSCORE I and EuroSCORE II in patients. It was 4.16% for EuroSCORE I and 0.86% according to EuroSCORE II. After discussion with the cardiothoracic surgeon, the patient was immediately planned by a senior thoracic and cardiovascular surgeon with 10 years of experience as a surgeon for mechanical mitral valve replacement (MVR) surgery, tricuspid valve repair (TVr), and LA thrombus evacuation. A multi-layered and easily detached thrombus size 4 × 3 × 2.2 cm was evacuated at the surgery. There was a dense brownish mass in the center and chewy consistency (Fig. 3). Microscopic examination of anatomical pathology showed extensive fibrin and extensive bleeding with mononuclear inflammatory cells and macrophages with a conclusion of a cardiac thrombus.

After surgery, the patient was evaluated by TTE. The results showed that the mechanical mitral valve was in good location and function (peak velocity 1.35 m/s; mean gradient 2.04 mmHg; VTI PrMv/VTI LVO 1.75; EOA 2.2 cm^2^; PHT 96 cm^2^). No thrombus in LA, negative LASEC. After the patient's condition stabilized, our patient was discharged eight days after surgery with oral furosemide 40 mg three times daily, spironolactone 25 mg once daily, and warfarin 2 mg once daily, also education to checks INR regularly and keep up cardiac rehabilitation. The patient feels relieved after the operation because the complaints of shortness of breath are much reduced. The patient was followed up for 1 year postoperatively with good results. The patient can carry out normal activities without any complaints.

## 3 Discussion

Management of MS should consider the appropriate timing based on the clinical characteristics and anatomy of the valve. In general, the indications for intervention are limited to patients with clinical significance moderate to severe MS (MVA planimetry <1.5cm2) [6]. Intervention with percutaneous mitral commissurotomy (PMC) can be considered. However, PMC is contraindicated when there is a thrombus in the LA, the degree of mitral regurgitation is more than mild, there are aortic and tricuspid valve disorders requiring surgery, or when there is an indication of coronary heart disease that requires bypass surgery [7].

MS management algorithm was described in the 2017 ESC on valvular heart disease [6]. In this case report, the patient has a planimetric MVA of 0.7cm2. The patient also has heart failure symptoms such as shortness of breath, swollen legs, and palpitations. However, the patient was contraindicated for PMC due to a thrombus in the LA. Therefore, the choice of therapy for MS in this patient is MVR surgery.

LA thrombus is common in patients with severe MS. Although we often find a LA thrombus in MS patients with atrial fibrillation, forming a free-moving, loose thrombus in the LA is rare [4]. Immediate treatment must be taken to avoid closure of the mitral inflow resulting in a sudden decrease in cardiac output. However, these free-moving LA thrombi are usually small, spherical, surrounded by an endothelial-like layer, and rarely cause systemic thromboembolism.

The finding of a large, freely moving thrombus in the LA has also been reported in several journals. Hange et al. reported the case of a 42-year-old woman who presented with complaints of shortness of breath during activities accompanied by a feeling of palpitations. Electrocardiography revealed atrial fibrillation. Echocardiography revealed severe MS and a freely moving LA mass. While waiting for the scheduled surgery, the patient had an embolic stroke. After MVR and thrombus evacuation, a mass weighing 12 g was obtained, with histopathology showing a thrombus [8]. Bansal and Kasliwal also published a case report of a patient with a large thrombus in the LA after mitral balloon valvuloplasty. The patient was then performed with an open mitral commissurotomy and evacuation of the thrombus [9]. Aoyagi et al. also published a case report of thrombus in the LA with atrial fibrillation but without MS. The cause of the thrombus is thought to be due to hypercoagulability because the patient has nephrotic syndrome [10].

**Fig. 4:**
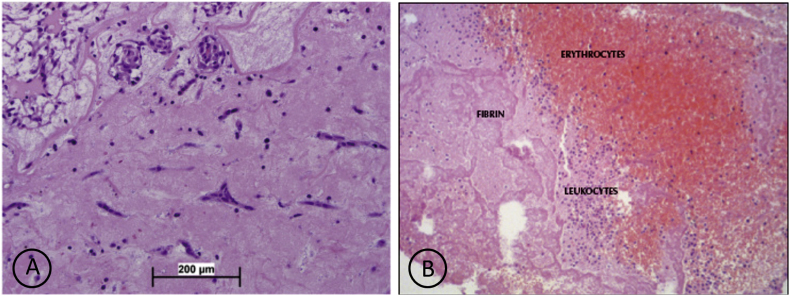
(A) Histopathological features in LA myxoma, we can find neoplastic cells proliferating in a myxoid stroma with scattered round, stellate or polygonal cells with dense irregular nuclei [10], (B) Histopathological features in LA thrombus, we can find erythrocytes, leukocytes, and fibrin [11].

LA myxoma is one of the differential diagnoses of LA thrombus. Histopathological examination is the gold standard for establishing the diagnosis. In LA myxoma, we can find neoplastic cells proliferating in a myxoid stroma with scattered round, stellate or polygonal cells with dense irregular nuclei (Fig. 4A) [11]. In contrast, the thrombus found erythrocytes, leukocytes, and fibrin (Fig. 4B) [12]. Echocardiography of the patient, in this case, revealed a freely moving ping-pong-like thrombus in the LA. The thrombus is round with firm walls resembling a myxoma-like formation. LA thrombus evacuation was performed on this patient. According to a thrombus, histopathological results found extensive fibrin formation and extensive bleeding with mononuclear inflammatory cells and macrophages.

Preparation for valvular heart disease surgery is, in principle, almost the same as preoperative heart surgery. When a patient is undergoing cardiac surgery, a comprehensive evaluation of the patient's condition and comorbidities is critical. Several things that need to be considered before surgery are the patient's history and physical examination and complete supporting examinations, both cardiac and extracardiac, so that complete and comprehensive preoperative data are obtained. This preoperative assessment is essential, one of which is to ensure the patient is in optimal condition during surgery so that it is hoped that the surgical outcome will be good with minimal complications [13].

Several scoring systems have been developed to help determine the risk stratification of perioperative heart surgery, including the European System for Cardiac Operative Risk Evaluation (EuroSCORE) and the score set by the Society of Thoracic Surgery Risk (STS score). There are two types of EuroSCORE, EuroSCORE I and EuroSCORE II, with the classification of risk groups divided into three risk groups, low risk (0–2%), moderate risk (3–5%), and high risk (>6%). STS is best at predicting patients undergoing aortic valve replacement (AVR) or coronary artery bypass graft (CABG) with simultaneous valve replacement. In contrast, EuroSCORE II is better at predicting patients undergoing CABG alone or mitral valve surgery alone [14]. We calculate EuroSCORE in our patient because the patient carried out MVR and TVr. The predicted mortality is a low-moderate risk, 4.16% according to EuroSCORE I and 0.86% according to EuroSCORE II.

## 4 Conclusions

LA thrombus is often seen in a patient with severe MS. TTE is still a reliable tool in diagnosing large mobile atrial thrombi, but histopathological examination is gold standard. Optimal preoperative preparation involves assessing preoperative risk stratification. Immediate thrombus evacuation and valve replacement will give good results and reduce the occurrence of systemic thromboembolism.

## Ethical Approval

Ethical Approval is not needed for a case report based on Dr. Soetomo General Hospital Ethical Committee.

However, the patient has signed an informed consent form for article publication.

## Sources of funding

The authors received no financial support for the research, authorship, and/or publication of this article.

## Author contributions

Erlangga Diasmara Hargiyanto: attending physician, data collection, writing the paper.

Ivana Purnama Dewi: writing the paper, formatted the manuscript for publication.

Budi Baktijasa Dharmadjati: supervision.

## Registration of research studies

This article is a case report.

## Guarantor

Erlangga Diasmara Hargiyanto.

## Provenance and peer review

Not commissioned, externally peer reviewed.

## Declaration of competing interest

The authors declare that there is no conflict of interest.
